# Chronic Respiratory Diseases in the Regions of Northern Russia: Epidemiological Distinctions in the Results of a National Population Study

**DOI:** 10.3390/ijerph14080841

**Published:** 2017-07-26

**Authors:** Marine H. Gambaryan, Svetlana A. Shalnova, Alexander D. Deev, Oxana M. Drapkina

**Affiliations:** Federal State Institution National Research Center for Preventive Medicine, Moscow 101990, Russia; sshalnova@gnicpm.ru (S.A.S.); adeev@gnicpm.ru (A.D.D.); odrapkina@gnicpm.ru (O.M.D.)

**Keywords:** chronic respiratory diseases, chronic obstructive pulmonary disease (COPD), regions of the Far North, smoking

## Abstract

The aim of the study is to investigate the epidemiological situation regarding chronic respiratory diseases in populations that inhabit different climatic–geographical regions of Russia, and to develop targeted programs for prevention of these diseases. Methods: (1) a comparative analysis of the standardized mortality data in Russia and other selected regions of the Russian North using the European standard for respiratory diseases, in a population aged 25–64; and (2) data from a randomized cross-sectional epidemiological study, with subjects from three different climatic-geographical regions of Russia. Results: (1) the respiratory disease-related mortality rates in the majority of Russian Northern regions were much higher compared to the national average. Although death rates from chronic lower respiratory diseases were higher among the Northern regions and in the whole of Russia relative to the countries of European Union (EU), the cause of death in the populations of the Northern regions tend to be lower respiratory infections and pneumonia; and (2) despite the absence of any significant differences in the prevalence of smoking, the prevalence of chronic respiratory diseases (COPD) is significantly higher in Far North Yakutsk compared to the other two regions in this study—Chelyabinsk and Vologda. The status of hyperborean had the highest chance of a significant contribution to COPD and cardiorespiratory pathology among all other risk factors. The results revealed a need for effective targeted strategies for primary and secondary prevention of chronic respiratory diseases for the populations of the Northern regions of Russia. Conclusions: The revealed regional distinctions regarding the prevalence of, and mortality from, chronic respiratory diseases should be taken into consideration when designing integrated programs for chronic non-communicable disease prevention in these regions.

## 1. Introduction

Chronic respiratory diseases (CRD)—chronic obstructive pulmonary disease (COPD) in particular—are serious social and economic burdens worldwide [1,2], including in the Russian Federation, where mortality due to COPD is one of the highest in the European region [3]. 

Respiratory diseases are the third leading cause of mortality in European Union (EU) countries, following cardiovascular diseases and malignant neoplasms: According to the Eurostat data, the standardized mortality ratio is 41.2 deaths per 100,000 of populations across all ages, with the majority of deaths in people over 65 years of age. Among all respiratory diseases, chronic lower respiratory diseases (CLRD) are the most frequent causes of death, followed by pneumonia [4]. 

Some work suggests that CLRDs, particularly chronic bronchitis (CB) and COPD, are independent risk factors of cardiovascular diseases (CVD). CB and COPD have a higher association with CVD than common smoking [5,6]. It has been shown that CB and COPD, as systemic inflammatory diseases, can increase the CVD development rate regardless of regular smoking [7]. Therefore, early detection and prevention of CB and COPD are important, not only for the prevention of their progression, but also for decreasing the risk of CVD.

The Russian North, with a territory comprising about 70 percent of Russia’s total area and with 25.3 million people or 17.8% of the national population, seems to face a much greater burden of CRD compared to Russia on average—which is already one of the highest in the European region due to the extreme climate and geographical and ecological conditions. The health of the population in the Russian North regions is largely influenced by the extreme climate conditions [8], as well as the difficult ecological conditions due to pollution from gas oil, aluminum, nickel, and other industries all over the region—the source of about 106,100 tons of pollutants annually, as well as exposure to occupational hazards at numerous industrial enterprises where the majority of the working population of the Northern regions is employed [9]. According to official statistics, the regions of the Russian Far North have concentrations of air pollutants that far exceed the maximum permissible emission standards, and 71% of the working population of Russian Far North is exposed to occupational hazards, far beyond occupational safety norms [10].

The major behavioral risk factor is smoking, which increases the risk of COPD and CVD, which is the world’s leading cause of mortality [11]. According to official statistics, the morbidity of respiratory diseases (RD) is, on average, 26.6% higher in the majority of the Russian Far North: Arkhangelskaya and Murmanskaya Oblasts, Komi and Karelia Republics, Republic of Sakha–Yakutia, and Yamal-Neneckiy Autonomous Okrug, compared to the country’s average. In addition, the rate of RD morbidity is 51.3% higher in Neneckiy Okrug of Arkhangelsk Oblast and 40.5% higher in Chukotskiy autonomous Okrug, compared to the Russian average [4]. Furthermore, according to official statistics, the rate of chronic respiratory diseases in the Far North population is more than twice the average rate in the Russian population [12]. It is also noted that the average age of death due to CRDs (ICD-10 Class X) in populations of several Northern regions is lower than that of the general Russian population. For example, the average age of death due to CRDs in the Chukotka Autonomous Okrug male population was 45.8 years in 2012 (with a national average of 64.7 years), according to the latest data. Such a pattern, however, is specific to the majority of the Northern regions, despite the fact that the population of the Russian Far North is relatively young—with the pension age being 4.7% lower than the general population of Russia [13].

In spite of an increase in CRD morbidity and mortality, and COPD morbidity and mortality in particular, up to 80% of patients remain undiagnosed [14], because both doctors and patients underestimate the disease [15]. These facts suggest that analysis of factors of CRD morbidity, as well as regional epidemiological distinctions of CRD, are essential for the prevention of these diseases. 

The aim of this study is to investigate the epidemiology of CRD in a population of economically active ages (25–64 years) inhabiting various climatic–geographical areas of the country, and to reveal the specific traits of development of these diseases in the Northern regions by using analytical methods in order to develop targeted integrated programs for prevention.

## 2. Materials and Methods

Two studies were performed to meet the set objectives:

1. *Analytical study.* Based on national medical statistics data, a comparative analysis of respiratory disease, age-standardized mortality rates in the general Russian population, and the populations of a number of Russian Far North regions, such as Murmansk and Arkhangelsk Oblasts, the Republic of Komi, Chukotka AO, and the Republic of Sakha (Yakutia), was performed, according to the European standard, in order to compare the results with European statistics. For this, the death rates due to respiratory diseases in the Russian regions were presented by official statistics as a ratio of deaths from RD to mid-year populations, calculated per 100,000 people. This ratio is then standardized using the direct method to represent what those death rates would have been if the population had the same age distribution as the standard European population. The sharing of respiratory diseases, in general mortality, was studied in the Northern territories in order to substantiate the priority areas for decreasing premature mortality.
2. *Cross-sectional epidemiological study*. Subjects aged 35–64 years (n = 3771, 81% response rate) from the representative sample of the national multicenter cross-sectional epidemiological study were included in the analysis. The study was performed according to a standardized protocol in the three regional capitals of the Russian Federation, with each belonging to a different climatic-geographical area: The midland region (Vologda, n = 1341), an industrial region in Ural (Chelyabinsk, n = 1305), and the Far North Region (Yakutsk, n = 1125). This study is a part of a multicenter epidemiological study that was aimed at investigating the epidemiological traits of chronic non-communicable diseases (NCDs)—by identifying common risk factors and evaluating the need in medical prevention of these diseases. The goal was to study CRD incidences. In particular, studying COPD and its relation to the common risk factors, and the region of inhabitance.

### 2.1. Sampling and Statistics

A systematic stratified multistage random sampling was employed (Kish method), using territorial principle on the basis of outpatient health clinics. First, a list of all outpatient health clinics in the region was established. Further random selection of the health facilities was performed. Then, from the randomly selected facilities, according the same principle as the random selection stage, the areas that were assigned to each of the clinic were selected. Within those areas, the ones that were assigned to the health sites were used to develop a list of addresses/households, and then addresses were randomly selected for this study. Finally, for the sample, one adult per family was randomly selected. 

Chi-squared tests and odds ratios (OR) were utilized to compare the data. Multivariate logistic regression was employed to analyze the regional associations of COPD and combined cardiorespiratory diseases, controlling for socio-demographics, behavioral factors, and comorbidities.

### 2.2. The Criteria Used for the Evaluation of Conditions and Risk Factors

Respiratory diseases and CLRD-ICD-10 codes J00–J99 were used for the respiratory diseases’ mortality rates and J40–47 for CLRD were used to analyze the data of the official medical statistics that were standardized according to the European standard, and to compare the data with the same statistics for European countries (based on World Health Organization (WHO) and EU data) [16].

COPD status was defined based on the population survey and positive answers to the question “Have you ever been told that you have an obstructive bronchitis/COPD/emphysema?” Taking into account that not all patients were diagnosed, and the possible deviation of the diagnostic criteria from the international criteria as recommended in Global Initiative for Chronic Obstructive Lung Diseases GOLD [17,18], the analysis was performed using two COPD status criteria—strict and extended.

Strict criteria COPD status was used for patients over 40 years old with any of the following conditions in their medical history: Chronic obstructive bronchitis, COPD, or emphysema; extended criteria COPD status was used for patients over 40 years old with chronic coughing for no less than three months a year and any of the following conditions in their medical history: Chronic obstructive bronchitis, COPD, or emphysema.

Cardiorespiratory conditions were set for patients with a combination of extended COPD status and cardiovascular condition, including arterial hypertension (AH) and/or angina, combined with CVD, in their medical histories, and/or signs of ischemic alteration in Electrocardiography ECG.

Smoking status (regular smoking) was taken into account if a patient smoked at least one cigarette per day.

Smoking intensity:

- light—up to 15 cigarettes a day;
- moderate—15–20 cigarettes a day;
- heavy—more than 20 cigarettes a day.

The data were processed and analyzed using the SAS statistical system (SAS Institute Inc., Cary, NC, USA, 1990).

## 3. Results and Discussion

**Figure 1:**
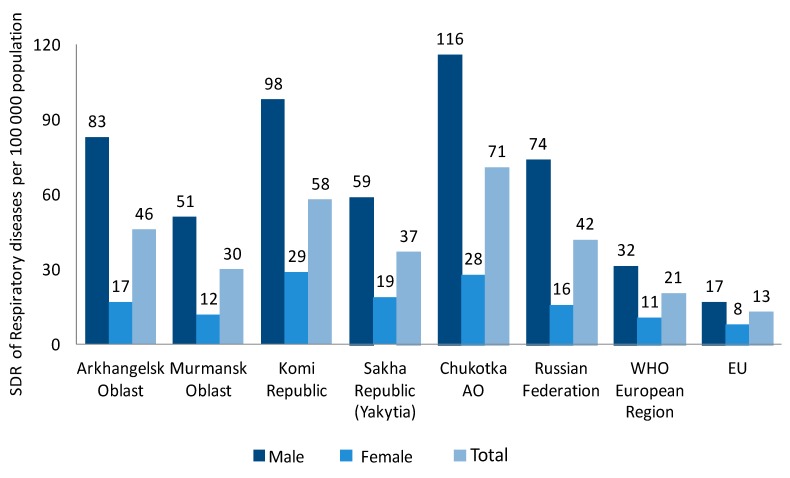
Standardized death rates (SDR) of respiratory diseases per 100,000 population in selected regions of the Russian North.

Comparative analysis of premature mortality parameters due to respiratory diseases shows that, in the majority of the Far North regions that were included in the analysis, the RD mortality rates were higher than the mortality rates in the general Russian population (41.9 per 100,000 people), which is already twice the RD mortality rate of the European regions (20.5 per 100,000) and three times the rate of the EU countries (12.5 per 100,000) (Figure 1).

The contribution of respiratory diseases to the mortality rates in the studied population (aged 25–64 years) (by the standardized criteria) varied from 3.0% in the Republic of Sakha to 5.1% in the Republic of Komi, while the general Russian value was 4.6%. The share of respiratory diseases in the EU premature mortality rate is 3.9% (in both males and females) [19].

Gender differences in premature mortality rates due to respiratory diseases should be noted. To illustrate, the data for the Arkhangelsk region shows that premature mortality due to CRD in males was five times higher than in females; in the general Russian population, this difference is 4.7 times, while in Europe, the difference is 2.1 times.

Respiratory diseases had the highest contribution in male mortality rates in the general Russian population, as well as in all of the studied regions. The highest difference was found in Chukotka autonomous Okrug, where the sharing of respiratory diseases in male premature mortality was two times higher than in females, with the lowest mortality in the Republic of Sakha (Yakutia)—a rate of 1.1 times higher in males than in females. The general Russian population was 1.6. On average, for the Russian population, the share of respiratory diseases in premature mortality was 5.1% in males and 3.2% in females, the biggest share of respiratory diseases in premature mortality was found in the Republic of Komi, in both males and females, 5.4% and 4.3%, respectively. 

**Figure 2:**
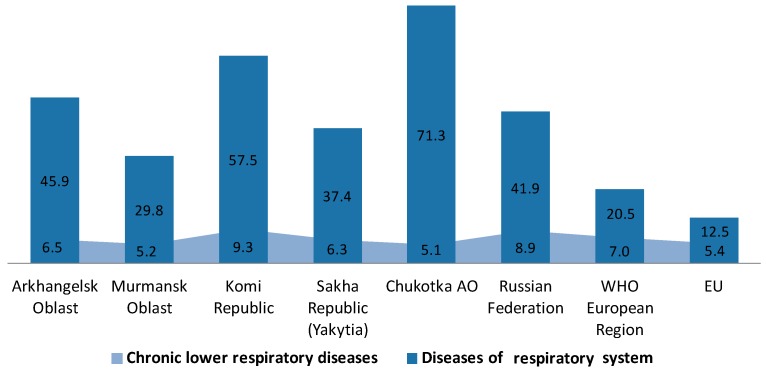
Standardized death rates of respiratory diseases and chronic lower RD per 100,000 in selected regions of the Russian North.

It should be noted that, while the mortality rates due to respiratory diseases in patients 25–64 years old in the studied regions were high, the CLRD mortality rate was relatively low and varied from 7.1% in the Chukotka Autonomous Okrug to 17.4% in Murmansk Oblast, with the average in Russia being 21.3%. For comparison, this parameter in the European region was 34%, and can reach as high as 43.5% in EU countries (Figure 2). At the same time, the mortality due to CLRD in most of the studied regions, and in Russia in general, was higher than in the EU, with exceptions in Murmansk Oblast and Chukotka Autonomous Okrug. According to the official statistics data, for the latter, people tended to die of respiratory diseases at an early age, before chronic respiratory conditions could develop. In those regions, the average age of death due to respiratory diseases in 2012 was 54.4, and among males—45.8 [13]. An additional analysis of the structure of mortality in the Far North Russian regions showed that in most cases, the mortality rates due to high respiratory diseases of this age group were due to lower respiratory infections and other unspecified respiratory diseases. Another possible reason for the relatively low CLRD mortality (represented mostly by chronic bronchitis and COPD) in the structure of high RD mortality in those regions was that CLRD is not always diagnosed or not diagnosed in time, and their exacerbations fall into the “acute lower respiratory infection” diagnosis.

Therefore, the conducted comparative analysis allows us to detect high premature mortality due to respiratory diseases in some Russian Far North regions of the country, especially the industrially developed ones (the Republic of Komi, and the Arkhangelsk Oblast) and regions consisting of mostly native population (Chukotka Autonomous Okrug). 

That being said, high and premature RD mortality was also registered in some midland and non-northern industrial regions, which calls for a more detailed investigation of the causes and interconnections, to help develop targeted disease prevention programs. In Vologda Oblast, in particular, the premature mortality rate was higher than in the general Russian population, and in 2010, the mortality rate was 61.4 per 100,000, with 112.1 for males and 28.3 for females. The standardized figure of premature mortality due to RD in the Chelyabinsk Oblast population was also higher than the average for the Russian population: 44.2 per 100,000 of the population, especially higher in males—80.5 (compared with 15.4 in females) per 100,000 people. It should be noted that Chelyabinsk Oblast had a very high CLRD mortality rate compared to the other studied regions—15.7 per 100,000 of the population, mostly due to the mortality rate in males (29.4), which is almost two times the Russian average. These results were the reason for including the mentioned regions in the CRD risk comparative epidemiological study.

Comparative analysis showed that 57.9% of the economically-active populations in Russia (the age group of 25–64 years as an example) eventually die from one of the three chronic NCDs with common risk factors: CVDs, neoplasms—including malignant neoplasms—and respiratory diseases, which makes RDs one of the three main causes of premature mortality in the Russian population, especially in the Northern regions.

The common risk factors for these diseases are either behavioral or environmental, with many of them potentially controllable. These three NCD groups, with common risk factors contributing the most to premature mortality in economically active populations in the Northern regions, which were higher than the average for the Russian population, especially in Arkhangelsk Oblast (approximately 91% in males) and Murmansk Oblast (in both males and females). 

The COPD incidence and risk factors study was conducted as a part of a multicenter epidemiological study, coordinated by the State Scientific Research Center for Preventive Medicine, based on representative samples of male and female subjects, 35–64 years old (n = 3771, 81% response rate) in the capitals of three regions of the Russian Federation: The cities of Yakutsk, Chelyabinsk, and Vologda. The populations of these regions had no definite differences in socio-demographical parameters, nor in the prevalence of smoking, as shown in Table 1.

**Figure 3:**
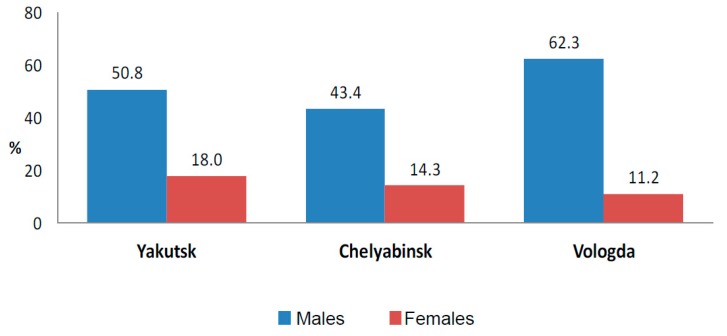
Prevalence of daily smoking in the studied regions.

Smoking prevalence in the general sample was 54.5% in males and 14.3% in females. The highest smoking prevalence in males was found in Vologda (62.3%), and the lowest in Chelyabinsk (43.4%). The prevalence in Yakutsk (50.8%) had no definite difference from other mainland and Ural regions. At the same time, the prevalence of smoking in female patients in Yakutsk appeared to be higher (18.4%) than in Chelyabinsk (14.3%) and Vologda (11.2%) (Figure 3).

The study showed the extended criteria for COPD prevalence in the sample population as 21.9–23.1% in male subjects and 21.1% in females, and the strict criteria COPD prevalence was 14.7–12.9% in males and 15.7% in females. COPD was diagnosed prior to the study, but only in half of the subjects (50.9%, *p* < 0.001). 

The largest COPD prevalence was found in Yakutsk—significantly higher than in the other regions, with 28.6–28.9% in males and 29.1% in females (*p* < 0.001).

**Figure 4:**
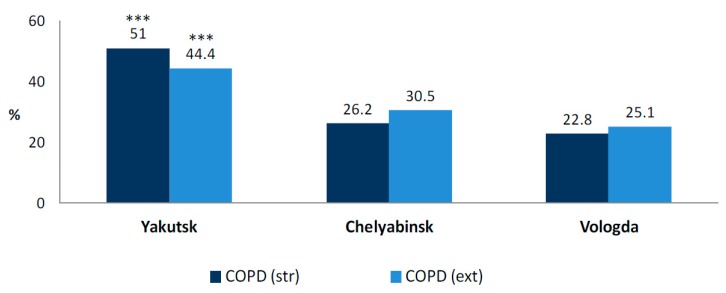
Prevalence of chronic obstructive pulmonary disease in the studied regions. *** *p* < 0.0001.

The extended criteria COPD rate was significantly higher in Yakutsk (44.4%) than in Chelyabinsk and Vologda (*p* < 0.001), and the strict criteria COPD prevalence formed more than a half of the studied population (51%, *p* < 0.001) (Figure 4).

The CB or COPD risk in the general sample analysis was significantly related to smoking and its intensity. The COPD risk in heavy smokers was 10.5 times higher in males (OR = 10.5, 95% CI: 5.4–19.2), and five times higher in females (OR = 5, 95% CI: 2.8–8.8) compared to non-smokers. COPD risk was also higher in moderate smokers (OR = 4.4, 95% CI: 2.9–6.7 and OR = 2.6, 95% CI: 1.8–3.8 in males and females, respectively) and light smokers (OR = 2.6, 95% CI: 1.8–3.8), compared to non-smokers. The risk was even higher in the recent quitters (OR = 3.06, 95% CI: 1.9–4.9) compared to those who had never smoked. 

As shown above, no significant differences in prevalence and intensity of smoking were found between the studied regions, but there was a significant relationship between the region of inhabitance and COPD. 

The association between the risk factors, COPD, and cardiorespiratory conditions were evaluated in our study. First, we measured the prevalence of socio-demographics, geographic, behavioral, and health risk factors in COPD extended, COPD strict, and combined extended and strict cardiorespiratory categories, and then evaluated if the geographical region of inhabitance may be a risk factor for COPD and cardiorespiratory conditions after adjusting for major socio-demographics, lifestyle, and comorbid risk factors.

Table 2 shows the prevalence of socio-demographics, geographic, and common behavioral and health risk factors in COPD extended, COPD strict, and combined cardiorespiratory extended and strict categories.

The analysis showed that a significant majority of COPD individuals are inhabitants of Yakutsk. Among them, the patient COPD strict category formed more than half of the examined population: 52.22 of men and 50.40 of women with COPD strict criteria lived in Yakutsk. The same was true for the combined cardiorespiratory categories, with the exception for women, whose shares in the categories of CRD and CVD extended, and CRD and CVD strict categories were higher in Chelyabinsk, though their shares in Yakutsk were still higher compared to Vologda—39.07% and 36.67% respectively. Another factor that was strongly prevalent in men in these categories was current smoking: 67.91% and 62.78% of male individuals of COPD (the extended and strict criteria), and 61.74% and 53.33% of the cardiorespiratory categories, respectively, were current smokers. 

Multivariate logistic regression analysis, controlled for age and gender, was conducted to investigate the associations of socio-demographic factors, health behavior factors with COPD, and cardiorespiratory conditions (Table 3). In addition to the socio-demographic characteristics and smoking status, factors such as drinking status, daily physical activity and body mass index (BMI), were included in the model. 

The analysis showed that the subjects—inhabitants of Yakutsk—had about three times the risk of COPD (both the extended and strict categories), and more than twice the risk of combined COPD and CVD in both criteria, compared with subjects from Vologda: (OR 2.8, 95% CI: 2.3–3.5), (OR 3.3, 95% CI: 2.6–4.2), (OR 2.1, 95% CI: 1.7–2.7), and (OR 2.3, 95% CI: 1.6–3.5), respectively (*p* < 0.001). Subjects from Chelyabinsk had about twice the risk of COPD and a combined cardiorespiratory condition compared to inhabitants from Vologda. This can be explained by the fact that Chelyabinsk, being a prominent industrial city in Ural, has similar difficulties of ecological conditions in terms of air pollution and occupational exposures.

Smoking was strongly associated with COPD and combined cardiorespiratory conditions, even those who had quit smoking still had a risk of COPD, 1.6–1.8 times higher than people who have never smoked. Smoking intensity increased this risk; heavy smokers had about seven times the risk for COPD by the extended criteria and four times for COPD by the extended criteria, compared to people who have never smoked: (OR 6.8, 95% CI: 4.4–10.2) and (OR 3.9 95% CI: 2.4–6.1) (*p* < 0.001). Moderate smokers are two to three times more likely to have COPD, compared to people who have never smoked (*p* < 0.001), and light smokers have a risk that is 1.5 times higher than people who have never smoked (*p* < 0.05).

Another factor that is strongly associated with the CRD, and combined CRD and CVD conditions appeared to be obesity with BMI > 33. These subjects have about 2–2.5 times the risk for COPD and combined cardiorespiratory conditions compared to those with normal BMI: (*p* < 0.0002). Borderline abnormal anxiety and depression, which are defined by the Hospital Anxiety and Depression scale (HADS), demonstrated some significant associations with the risk of CRD and combined CRD and CVD conditions in both extended and strong criteria: (*p* < 0.05). 

Further conducted multivariate stepwise regression analysis showed significantly higher risks of COPD and combined cardiorespiratory diseases in the Yakutsk and Chelyabinsk populations compared to Vologda, in both males and females. The occurrence of COPD extended was 2.2 times higher in males (OR = 2.2, 95% CI: 1.6–2.98; *p* < 0.001), and 3.5 times in females (OR = 3.5, 95% CI: 2.7–4.6; *p* < 0.001) in Yakutsk compared to Vologda. The risk of combined cardiorespiratory diseases was 1.7 higher in male subjects (OR = 1.7, 95% CI: 1.3–2.2; *p* < 0.001), and 2.5 times in females (OR = 2.5, 95% CI: 2.1–3.2; *p* < 0.001) in Yakutsk compared to Vologda.

Inhabitance of the Far North regions made the largest contribution to the risk of chronic respiratory and cardiorespiratory diseases, supplanting and surpassing all other risk factors for these groups of diseases by a prognostic value derived from the multivariate model, including light and moderate smoking, and smoking in the past. Only heavy smoking could have been compared by its contribution to the risk of the mentioned diseases. 

Thus, the prevalence of high respiratory diseases and mortality rates in the Far North region may be largely due to climatic, environmental, and ecological factors. That being said, it should also be highlighted that the negative effects of smoking, as a traditional COPD risk factor, are significantly potentiated by complex environmental factors, such as difficult climatic–geographical conditions, ecological particularities of inhabitance, and working environment conditions. Combined with these factors, smoking remains one of the top priorities of controllable causes of COPD incidence and premature mortality in the population of Northern regions, especially in the regions with higher ecological burden and developed industry. It should also be highlighted that the task of integrated prevention of the mentioned diseases does not belong solely to the healthcare system; it should also be the responsibility of state authorities and inter-sector efforts, which are especially important in Northern Russia, given the complexity of the harmful factors that are found there.

### Potential Limitations of the Study

In this study, it was not possible to fully separate the roles of the climate factors, ecological conditions, and occupational hazards, in terms of the risk of COPD and other related conditions. This was because these factors are most often presented together as a complex system that can contribute to harmful effects. However, when comparing a Northern region with an industrial one, especially one with a difficult ecological situation in terms of the risk of COPD and other related conditions, the contributions of climatic factors are evident. 

The diagnosis of COPD and other related conditions in the cross-sectional study were self-reported, which can be seen as a threat to validity, and hence, was considered another limitation to this study. However, several previously-conducted studies have confirmed the validity and analysis of self-reported data [20,21]. Secondly, our results matched with the findings of the cross-sectional population-based epidemiological study of The Global Alliance against Respiratory Diseases (GARD) among adults from 12 regions of the Russian Federation, which has revealed a prevalence of self-reported CB as 22.2%, and a prevalence of COPD based on spirometry in patients with respiratory symptoms as 21.8% [22]. These give us a certain degree of confidence about the reliability of our data. 

## 4. Conclusions

A comparative study of premature mortality parameters that were standardized by age allowed us to determine regional peculiarities, such as a high mortality due to respiratory diseases compared to the national average, and high mortality due to lower respiratory tract infections, which should be taken into account when developing integrated programs for NCD prevention.

A study of COPD prevalence and risk factors, conducted as a part of the multicenter epidemiological study, found a significantly higher COPD prevalence in the Far North region Yakutsk, compared to the other two regions—Chelyabinsk and Vologda, while smoking prevalence was similar in all three regions. Inhabitance of the Far North region also made the largest contribution to the risk of chronic respiratory and cardiorespiratory diseases, supplanting and surpassing all other factors in terms of statistical value. 

These results suggest that further studies are needed in: (1) investigating the role and the mechanisms of climate conditions, as well as occupational and ecological factors, leading to a higher risk for COPD patients, and combine these factors to “with” and “without” smoking; and (2) development of effective strategies for COPD prevention in populations who are exposed to those conditions. 

The results must be taken into account when developing NCD prevention programs for these regions. Comprehensive measures aimed at the harmful factors specific to the Northern regions should be included in NCD prevention programs, alongside smoking decrease and other NCD behavioral risk factors management. These measures that are aimed at decreasing the share of preventable risk factors on an individual level and increasing the precision of targeted prevention are necessary to decrease CRD and NCD prevalence and mortality in the general population of the country.

## Figures and Tables

**Table 1:** Characteristics of the studied populations in the three different cities.

Variables (%)	Total (n = 3771)		Yakutsk (n = 1125)		Chelyabinsk (n = 1305)		Vologda (n = 1341)	
	m	f	m	f	m	f	m	f
**Sex**	36.9	63.1	38.7	61.3	28	72	41.5	58.5
**Age (M ± SD)**	48.8 ± 8.2	48.6 ± 8	47.8 ± 8.4	48.6 ± 8	48.3 ± 9	50.6 ± 8.9	49.8 ± 7.5	49.4 ± 7.4
**Marital status**								
Married	73.5	56.4	77.5	57	70.3	48.2	71.8	62.9
Single	26.5	43.6	22.5	43	29.7	51.8	28.3	37.1
**Education**								
Primary	2.9	4.1	3.7	3.2	2.8	6	2.3	3.3
Secondary	67.4	60.1	57.8	49.6	60.5	62.1	79.1	67.7
Higher (University)	29.4	35.8	38.6	47.2	36.7	31.9	18.7	29
**Employment**								
Employed	78.2	74.4	74.7	71.5	85	71.5	77.9	78.9
Unemployed	21.8	27.7	25.3	28.5	15	28.5	22.1	21.1
**Smoking**								
Never smoked	28.9	80.4	33.7	77.5	32.2	78.7	23.5	84.5
Occasional smokers	16.7	5.3	15.1	4.6	24.5	7.3	14.3	4.3
Regular smokers	54.5	14.3	51.2	17.9	43.4	14	62.2	11.2
**Smoking intensity**								
Light	33.6	6	34.5	7.5	24.1	5.8	37.2	4.8
Moderate	17	5.9	13.1	7.6	14.3	6.1	21.3	4.3
Heavy	4	2.3	3.7	2.8	4.9	2	3.7	2.1

Note: m—male, f—female.

**Table 2:** Prevalence of risk factors in different chronic obstructive pulmonary disease (COPD), and combined COPD and cardiovascular diseases (CVD) categories.

Variables	% in All		% in COPD Extended		% in COPD Strict		% in COPD and CVD Extended		% in COPD and CVD Strict	
	n = 3771		n = 824 (21.85%)		n = 553 (14.66%)		n = 619 (16.41%)		n = 210 (5.57%)	
	m	f	m	f	m	f	m	f	m	f
**Gender**	36.91	63.09	32.55	67.45	37.16	62.84	28.57	71.43	38.96	61.04
**Age (mean, SE)**	48.82 (0.22)	49.49 (0.17)	49.35 (0.26)	50.01 (0.19)	49.95 (0.33)	50.16 (0.23)	49.97 (0.29)	50.62 (0.22)	50.80 (0.54)	51.16 (0.34)
**Region**										
Chelyabinsk	20.55	30.94	19.94	37.18	13.89	32.17	24.78	42.42	21.67	47.33
Vologda	44.25	36.44	36.45	17.89	33.89	17.43	39.13	18.51	36.67	16
Yakutsk	35.20	32.62	43.61	44.93	52.22	50.40	36.09	39.07	41.67	36.67
χ^2^			14.205	97.904	26.49	87.005	4.102	66.794	1.598	33.076
*p*			0.001	0.001	0.001	0.001		0.001		0.001
**Marital status**										
Married/partnership	73.5	56.4	71.56	51.69	71.51	52.55	73.8	48.33	70	45.33
Single	26.5	43.6	28.44	48.31	28.49	47.45	26.2	51.67	30	54.67
χ^2^			0.778	5.810	0.407	2.701	0.015	12.389	0.388	8.006
*p*				0.016				0.001		0.005
**Education**										
Primary	2.9	4.1	4.98	5.37	6.67	4.83	5.22	6.17	5	6
Secondary	67.7	60.1	71.96	60.04	68.33	57.64	70	61.44	61.67	58
Higher (University)	29.4	35.8	23.05	34.59	25	37.53	24.78	32.39	33.33	36
χ^2^			13.253	2.679	11.713	1.335	7.458	6.413	1.662	1.490
*p*			0.001		0.003		0.024	0.041		
**Employment**										
Unemployed	21.8	25.6	34.27	29.03	37.78	29.49	34.35	34.19	48.33	40.67
Employed	78.2	74.4	65.73	70.97	62.22	70.51	65.65	65.81	51.67	59.33
χ^2^			38.285	4.034	31.119	3.598	25.608	18.218	25.987	19.211
*p*			0.001	0.045	0.001	0.058	0.001	0.001	0.001	0.001
**Smoking status**										
Non-smoker	28.9	80.4	14.95	70.78	15	73.99	18.7	73.52	16.67	77.33
Ex-smoker	16.67	5.1	17.13	5.37	22.22	5.9	19.57	3.86	30	3.33
Current smoker	54.5	14.3	67.91	23.86	62.78	20.11	61.74	22.62	53.33	19.33
χ^2^			41.950	48.683	20.249	13.176	13.968	27.536	9.941	4.298
*p*			0.001	0.001	0.001	0.001	0.001	0.001	0.007	
**Alcohol consumption**										
Non drinker	16.8	25.2	18.38	24.45	25	26.27	16.52	24.42	25	23.33
Occasional drinker	42.8	57	45.48	56.66	46.11	53.35	48.26	58.87	51.67	60
Light drinker	28.6	14.8	25.55	13.92	20.56	14.75	24.35	12.6	15	12.67
Moderate drinker	7.3	2.3	5.92	3.58	5	4.29	6.96	3.08	3.33	2
Heavy drinker	4.53	0.7	4.67	1.39	3.33	51.34	3.91	1.03	5	2
χ^2^			3.706	10.244	15.542	12.113	3.920	4.214	9.061	5.234
*p*				0.037	0.004	0.017			0.06	
**Physical activity**										
No PA < 30 min walk	31.47	25.81	33.02	27.04	30	25.20	33.48	24.94	21.67	22.67
Any PA ≥ 30 min walk	52.23	58.13	43.61	53.48	42.78	54.69	43.48	52.44	43.33	50.67
PA unknown	16.31	16.06	23.36	19.48	27.22	19.63	23.04	22.62	35.00	26.67
χ^2^			18.99	7.413	18.778	5.484	12.059	15.211	16.298	13.371
*p*			0.001	0.025	0.001	0.064	0.002	0.001	0.001	0.001
**BMI**										
BMI ≤ 21	5.46	6.4	8.10	4.77	7.78	5.36	6.96	3.6	8.33	5.33
21 < BMI ≤ 25	31.2	28	31.46	21.67	25	22.52	27.39	17.22	21.67	18
25 < BMI ≤ 29	41	29.8	35.2	27.83	40	29.22	33.91	26.99	40	25.33
29 < BMI ≤ 33	17.2	20.4	17.13	21.87	17.78	20.11	21.30	23.65	18.33	20.67
BMI > 33	5	14.6	8.10	21.87	9.44	20.91	10.43	25.96	11.67	28.67
BMI unknown	0.3	0.8	0	1.99	0	1.88	0	2.57	0	2
χ^2^			18.213	47.933	13.648	24.220	25.943	88.3821	8.706	31.768
*p*			0.003	0.001	0.001	0.001	0.001	0.001		0.001

**Table 3:** The association of the risk factors with COPD/cardiorespiratory categories that based on the results of multivariate logistic regression analysis.

Variables	CRD Extended	CRD Strict	CRD and CVD Extended	CRD and CVD Strict
	OR (95% CI)	OR (95% CI)	OR (95% CI)	OR (95% CI)
**Age**	1.030 (1.019–1.042) ***	1.030 (1.017–1.044) ***	1.050 (1.036–1.063) ***	1.047 (1.026–1.068) ***
**Gender**				
Male	1	1	1	1
Female	1.088 (0.884–1.340)	1.418 (1.112–1.809)	0.940 (0.744–1.188)	1.177 (0.802–1.729)
**Region**				
Vologda	1	1	1	1
Chelyabinsk	2.053 (1.642–2.565) ***	1.618 (1.236–2.117) ***	1.992 (1.572–2.524) ***	2.006 (1.352–2.975) ***
Yakutsk	2.837 (2.299–3.501) ***	3.278 (2.571–4.179) ***	2.119 (1.663–2.698) ***	2.349 (1.583–3.486) ***
**Marital status**				
Married/partnership	1	1	1	1
Single	1.097 (0.923–1.303)	1.064 (0.873–1.296)	1.136 (0.939–1.375)	1.230 (0.913–1.657)
**Education**				
Primary	1.224 (0.819–1.829)	1.305 (0.836–2.037)	1.264 (0.827–1.930)	1.156 (0.606–2.206)
Secondary	1	1	1	1
Higher (University)	0.922 (0.765–1.111)	1.045 (0.846–1.291)	1.011 (0.820–1.245)	1.443 (1.046–1.990)
**Employment**				
Employed	1	1	1	1
Unemployed	1.130 (0.893–1.429)	1.087 (0.828–1.426)	1.147 (0.888–1.482)	1.397 (0.948–2.058)
**Smoking status**				
Never smoked	1	1	1	1
Ex-smoker	1.558 (1.161–2.092) *	1.767 (1.272–2.455) **	1.287 (0.923–1.794)	1.476 (0.893–2.437)
Light smoker	1.906 (1.472–2.469) ***	1.393 (1.019–1.905) *	1.412 (1.044–1.909) *	1.270 (0.765–2.109)
Moderate smoker	3.330 (2.538–4.368) ***	2.330 (1.698–3.198) ***	3.000 (2.227–4.043) ***	1.981 (1.205–3.257) *
Heavy smoker	6.719 (4.425–10.201) ***	3.851 (2.447–6.060) ***	4.840 (3.135–7.472) ***	3.013 (1.543–5.885) **
**Alcohol consumption**				
Non drinker	0.936 (0.714–1.228)	1.029 (0.753–1.406)	1.025 (0.753–1.394)	1.254 (0.752–2.091)
Occasional drinker	1.030 (0.821–1.291)	0.992 (0.758–1.298)	1.182 (0.915–1.528)	1.428 (0.919–2.218)
Light drinker	1	1	1	1
Moderate drinker	1.055 (0.682–1.633)	1.324 (0.800–2.194)	1.153 (0.707–1.880)	0.823 (0.305–2.218)
Heavy drinker	1.224 (0.701–2.137)	1.103 (0.545–2.232)	0.982 (0.507–1.902)	2.161 (0.830–5.627)
**Physical activity**				
Any PA ≥ 30 min walk	1	1	1	1
No PA < 30 min walk	1.171 (0.967–1.418)	0.995 (0.794–1.246)	1.209 (0.974–1.501)	0.982 (0.681–1.417)
PA unknown	1.120 (0.839–1.495)	1.291 (0.925–1.801)	1.141 (0.832–1.566)	1.555 (0.958–2.524)
**BMI**				
BMI ≤ 21	1.127 (0.781–1.627)	1.189 (0.777–1.818)	1.100 (0.706–1.715)	1.421 (0.731–2.760)
21 < BMI ≤ 25	1	1	1	1
25 < BMI ≤ 29	0.975 (0.786–1.210)	1.163 (0.904–1.496)	1.136 (0.883–1.461)	1.175 (0.775–1.782)
29 < BMI ≤ 33	1.081 (0.846–1.382)	1.117 (0.837–1.492)	1.497 (1.138–1.970) *	1.235 (0.780–1.953)
BMI > 33	1.772 (1.334–2.337) ***	1.810 (1.321–2.478) ***	2.565 (1.899–3.465) ***	2.502 (1.582–3.959) ***

Note. * *p* < 0.05; ** *p* < 0.01; *** *p* < 0.001.
